# Human-Mediated Gene Flow Contributes to Metapopulation Genetic Structure of the Pathogenic Fungus *Alternaria alternata* from Potato

**DOI:** 10.3389/fpls.2018.00198

**Published:** 2018-02-15

**Authors:** Jing-Wen Meng, Dun-Chun He, Wen Zhu, Li-Na Yang, E-Jiao Wu, Jia-Hui Xie, Li-Ping Shang, Jiasui Zhan

**Affiliations:** ^1^Fujian Key Laboratory of Plant Virology, Institute of Plant Virology, Fujian Agriculture and Forestry University, Fuzhou, China; ^2^State Key Laboratory of Ecological Pest Control for Fujian and Taiwan Crops, Fujian Agriculture and Forestry University, Fuzhou, China

**Keywords:** *Alternaria alternata*, metapopulation genetic structure, admixture, isolation-by-distance, human-mediated gene flow, neutral evolution, microsatellite marker

## Abstract

Metapopulation structure generated by recurrent extinctions and recolonizations plays an important role in the evolution of species but is rarely considered in agricultural systems. In this study, generation and mechanism of metapopulation structure were investigated by microsatellite assaying 725 isolates of *Alternaria alternata* sampled from potato hosts at 16 locations across China. We found a single major cluster, no isolate-geography associations and no bottlenecks in the *A. alternata* isolates, suggesting a metapopulation genetic structure of the pathogen. We also found weak isolation-by-distance, lower among than within cropping region population differentiation, concordant moving directions of potato products and net gene flow and the highest gene diversity in the region with the most potato imports. These results indicate that in addition to natural dispersal, human-mediated gene flow also contributes to the generation and dynamics of the metapopulation genetic structure of *A. alternata* in China. Metapopulation structure increases the adaptive capacity of the plant pathogen as a result of enhanced genetic variation and reduced population fragmentation. Consequently, rigid quarantine regulations may be required to reduce population connectivity and the evolutionary potential of *A. alternata* and other pathogens with a similar population dynamics for a sustainable plant disease management.

## Introduction

Species can be structured geographically into either a series of genetically unconnected subpopulations (island model, Polz et al., 2013; Teixeira and Barker, 2016), many marginal subpopulations one direction connected to a large refuge population (island refuge model), an undivided single population (panmictic model, Kerr et al., 2006; Jiang et al., 2010), or arrays of local subpopulations interconnected by regular extinction and recolonization events (metapopulation model, Harrison and Hastings, 1996; Arnaud, 2003; Bay et al., 2008). How populations are spatially aggregated can have a profound impact on the population dynamics and evolutionary landscapes of species. In the field of plant pathology, understanding the way in which pathogen populations are genetically structured over spatial scales and evolutionary forces responsible for the generation and maintenance of the population genetic structures is important in formulating effective and sustainable plant disease management (Zhan et al., 2014). For example, regional resistance deployment may be effective to control plant diseases caused by pathogens spatially unconnected while not appropriated to control those caused by pathogens distributed in panmixia and/or metapopulation structure (McDonald and Linde, 2002).

Many plant pathogens in agricultural ecosystems are expected to be spatially aggregated in a pattern of metapopulation genetic structure. Metapopulations are generated by an interaction between recurrent local extinctions and recolonizations with high population turn-over (Hanski, 2011). Modern agriculture accelerates the local population turn-over of plant pathogens with extinction and recolonization events resulting from agricultural seasonality, deployment of host resistances and agrochemicals, changes in cultivation systems and global trade in agricultural products (Zhan et al., 2014, 2015). For example, due to epidemic cycles of diseases driven by annual crop growth dynamics and crop rotations, many plant pathogens (Stukenbrock and McDonald, 2008; Ali et al., 2014) do not survive on site and new epidemic cycles are usually triggered by natural recolonizations from other subpopulations or as a consequence of accidental human introduction. Crop rotations, seasonal fallows, replacement of previously widely-adapted host genotypes and/or introduction of newly-developed pesticides create unavoidable conditions driving the extinction of existing pathogen subpopulations and their subsequent replacement by founder subpopulations through migration from other extant subpopulations in the general area.

Population recolonizations can be achieved through movements (dispersals) of reproductive units of species from one or more nearby subpopulations either through an island (Lande, 1992) or stepping-stone model (Kimura and Weiss, 1964). In agricultural ecosystems, both natural and human-mediated movements can contribute to the recolonization events of plant pathogens. The extent and distance of natural movement are determined mainly by interactions among the dispersal mechanisms of the plant pathogens involved, climatic conditions and the landscape characteristics of the surrounding environment. Human-mediated movement can greatly facilitate the dispersal of plant pathogens, particularly when the pathogen species concerned have limited natural dispersal ability or geographic barriers such as rivers and mountains prevent natural movement to occur. With ever-increasing anthropogenic activities associated with the globalization of agricultural products and human migration etc., human-mediated movement is expected to play an increasingly important role in the generation and maintenance of metapopulation genetic structure of plant pathogens.

In recent decades, the study targeting to understand metapopulation genetic structures and dynamics of species has become a central theme and common framework in evolutionary ecology (Hanski, 1998; Hanski and Mononen, 2011; Burdon and Thrall, 2014; Castorani et al., 2017). However, the majority of the study was focused on natural ecosystems. Experimental studies on the metapopulation genetic structure of plant pathogens particularly the contribution of human-mediated dispersal to the generation ion and maintenance of metapopulation genetic structure of the plant pathogens in agricultural ecosystems are limit though they are highly relevant to sustainable disease management. Furthermore, frequent pathogen extinction events after host crop plants are harvested or replaced and the repeated formation of founder subpopulations associated with recolonization events in agricultural ecosystems allow us to study the genetic structure of metapopulations and its impacts on the evolution of species with a greater level of control than what may be possible in many natural ecosystems. Though distinguishing the effects of natural and human-mediated dispersal on the metapopulation genetic structure of species is difficult and rarely attempted (but see Kerr et al., 2006; Orozco-Ramírez et al., 2016), it could be achieved by undertaking a hierarchical analyses of genetic diversity and gene flow at different spatial scales (Bataille et al., 2011; Cornille et al., 2015; Kameyama et al., 2017). Gene flow mediated by natural dispersal alone is expected to create a strong isolation-by-distance among subpopulations in neutral markers and the direction of gene flow should consist with that of vector movement. On the other hand, gene flow driven by anthropogenic activities may generate a different pattern of spatial population genetic structure characterized by higher genetic similarity among long- than short-distance subpopulations and a weak or no isolate-by-distance. The direction of human-mediated gene flow can be different to that of vector movement.

In this study, we used potato-*Alternaria alternata* plant-pathogen system to test the hypotheses that many plant pathogens in agricultural ecosystems are spatially aggregated as metapopulation structure and anthropogenic activities play an important role on the population genetic structure of plant pathogens. *A. alternata* is an opportunistic haploid fungal pathogen causing leaf spots, blights, and other diseases on many plant species (Woudenberg et al., 2013) though host specificity has been documented in some studies (Elena, 2006; Woudenberg et al., 2015). The diseases are polycyclic and epidemics can rapidly develop under favorable environmental conditions. In potato, *A. alternata* causes early blight, one of increasingly important diseases of potato crop, forming symptoms of dark-colored spots with a pattern of concentric rings on leaves. The pathogen has a wide geographic distribution and can be dispersed by rain-splash, wind, and infected plant materials (Escuredo et al., 2011). It could survive for several months on potato debris in fields under dry and mild winters. Though cryptic sexual reproduction has been inferred by molecular analyses of genetic variation, mating type distribution, and phylogenetic trees (Meng et al., 2015a), no sexual reproductive structures have been observed yet either in the laboratory or fields. Therefore, field epidemics are believed to be initiated by primary inoculum originated from conidiospores or mycelium in the infected seed tubers or plant debris remained in the fields (Rotem, 1994; Batista et al., 2006).

The specific objectives of the current study were (i) to investigate the spatial distribution of genetic variation in *A. alternata* populations and through this (ii) to infer the contribution of human-mediated gene flow to the spatial population genetic structure of *A. alternata*. To achieve this goal, we assayed 725 *A. alternata* isolates sampled from 17 subpopulations across the main potato production areas in China with eight neutral SSR markers and evaluated “isolation-by-distance” to determine how geographic barriers may affect the gene flow in the pathogen populations. We then inferred the contribution of human-mediated dispersal on the population genetic structure of the pathogen by comparing the extent of gene flow (*Nm*) within and among potato cropping regions estimated from a classical island model (Lande, 1992), and the direction of potato seed tuber movement and net gene flow calculated by a coalescent framework (Beerli and Felsenstein, 2001).

## Materials and methods

### Fungal collections and microsatellite genotyping

**Figure 1:**
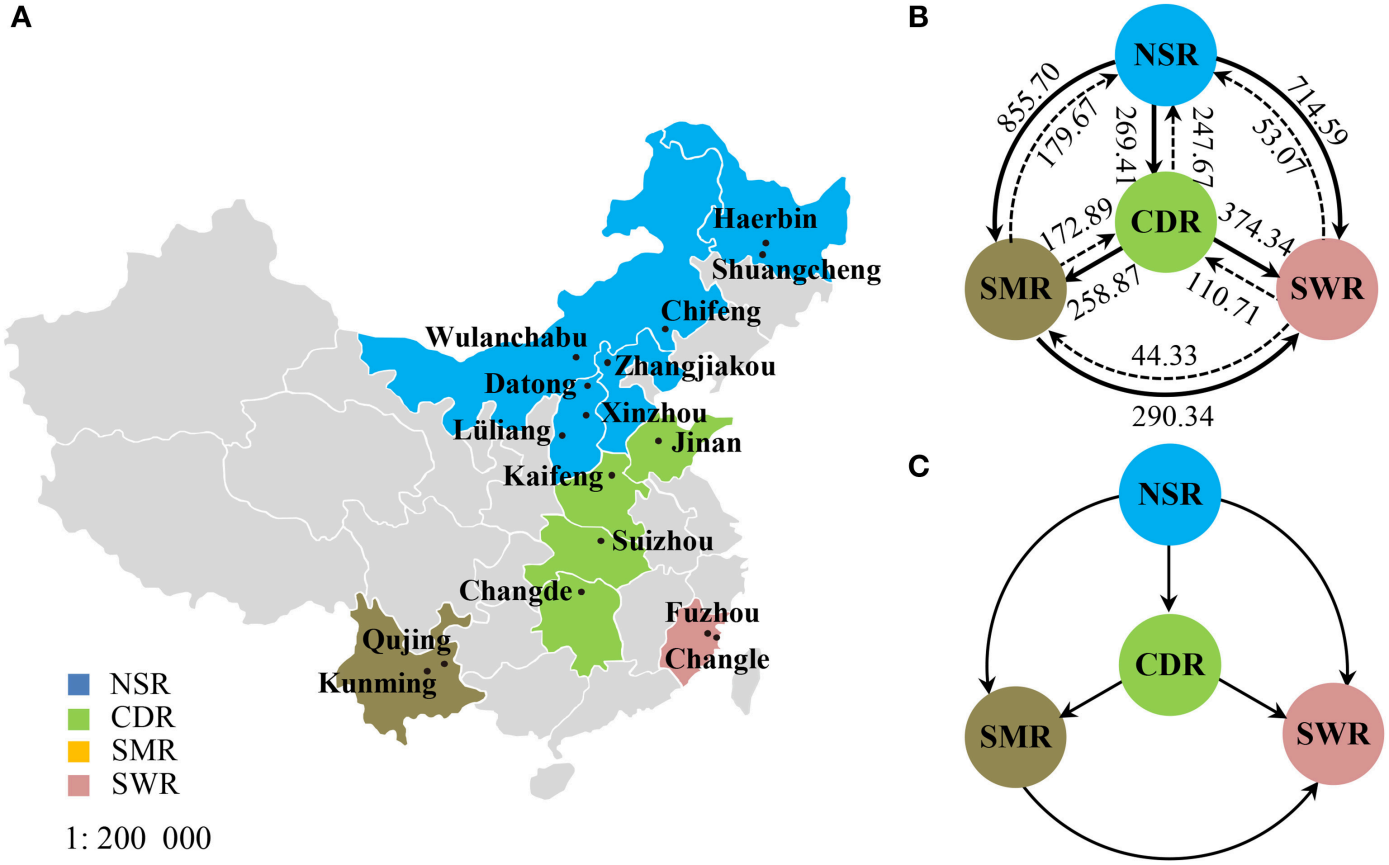
Geographic locations and gene flow in the *Alternaria alternata* subpopulations from China. **(A)** Map showing the geographic locations of 17 field subpopulations in the four potato cropping regions; **(B)** Estimated number of migrants in each generation among the four cropping regions; **(C)** The net migrants among four cropping regions.

A total of 725 *A. alternata* isolates collected from 17 fields across four cropping regions in China (Figure 1A, Table 1) between 2011 and 2013 was previously genotyped with eight pairs of microsatellite markers by the group (Meng et al., 2015a,b). KMG1 and KMG2 were the two populations sampled from the same field but different year. The detailed information on collection, isolation, DNA extraction, and microsatellite genotyping of the fungal pathogen can be found in the two publications. Briefly, potato leaves with the clear symptoms of early blight were sampled randomly at >100 cm intervals and only one infected leaf was collected from each potato plant. The infected leaves were placed separately in sandwich bags to prevent cross-infection and transferred to the laboratory in ice boxes. To isolate the pathogen, the infected leaves were rinsed with distilled water for 15 s, surface sterilized with 75% alcohol for 60 s and then incubated at 25°C on 1% water-agar medium. After 24 h, a single conidium was taken from each infected leaf, transferred to a potato dextrose agar (PDA, potato 200 g/L, glucose 20 g/L, agar 20 g/L) plate and incubated at 25°C for 2 weeks. The resultant isolates were purified three times by repeatedly transferring a single conidium to fresh PDA plates. Mycelia after the third purification were harvested for DNA extraction. Genomic DNA was extracted using a plant gDNA kit (Promega Biotech. Co. LTD., Beijing) and amplified with SSR primers in a total reaction volume of 25 μL using a 2720 thermal cycler (Applied Biosystems, Foster City, California). The primers were fluorescently labeled at the 5′ end and SSR amplifications were carried out using the following program: initially held at 95°C for 5 min; followed by 35 cycles of 94°C for 30 s, 57°C for 30 s, 72°C for 1 min; and ended with an extension step at 72°C for 5 min. Amplification sizes were determined using an ABI 3730XL automated DNA sequencer (Applied Biosystems, California) in which a DNA size ladder was included in each of the samples. Because both *A. solani* and *A. alternata* can induce potato early blight (Boiteux and Reifschneider, 1994; Weber and Halterman, 2012), all isolates were checked morphologically under a light microscope and molecularly by PCR amplification of ITS regions with primers 5′-TCCGTAGGTGAACCTGCGC-3′ and 5′-TCCTCCGCTTATTGATATGC-3′ to confirm their identity as *A. alternata* (Meng et al., 2015a,b) before SSR genotyping.

**Table 1:** Sample size and genetic variation in the *Alternaria alternata* subpopulations sampled from 17 fields across four potato cropping regions in China.

**Cropping region**	**Population**	**Code**	**Year**	***N***	**Allele (Private allele) number**	**Genotypes**	**Gene diversity**	**Shannon index**
CDR	Chengde	CGD	2012	24	3.38 (0.00)	23	0.50	1.00
	Jinan	JNN	2012	70	4.00 (0.00)	50	0.35	0.89
	Kaifeng	KFG	2012	80	3.88 (0.13)	50	0.35	0.87
	Suizhou	SZH	2012	52	4.00 (0.00)	37	0.39	0.91
Subtotal				226	4.88 (0.13)	160	0.40	0.88
NSR	Chifeng	CIF	2012	80	4.50 (0.13)	52	0.33	0.87
	Datong	DAT	2013	26	3.25 (0.00)	17	0.40	1.00
	Harbin	HRB	2012	76	3.75 (0.00)	48	0.31	0.85
	L liang	LLG	2013	9	2.50 (0.00)	7	0.32	0.86
	Shuangcheng	SHC	2012	42	4.13 (0.00)	35	0.36	0.93
	Wulanchabu	ULC	2012	23	2.63 (0.00)	16	0.25	0.85
	Xinzhou	XZH	2013	13	2.13 (0.00)	10	0.25	0.86
	Zhangjiakou	ZJK	2012	70	4.63 (0.25)	47	0.32	0.87
Subtotal				339	5.75 (0.50)	232	0.35	0.73
SMR	Kunming1	KMG1	2011	86	5.13 (0.50)	51	0.36	0.87
	Kunming2	KMG2	2012	7	2.13 (0.00)	5	0.33	0.87
	Qujing	QJG	2011	8	2.50 (0.00)	8	0.34	1.00
Subtotal				101	5.63 (0.50)	64	0.39	0.87
SWR	Changle	CHL	2011	11	3.50 (0.00)	8	0.56	0.84
	Fuzhou	FZH	2011	48	4.88 (0.13)	32	0.64	0.90
Subtotal				59	4.88 (0.38)	40	0.66	0.85
Total				725	7.25 (7.25)	253	0.42	0.78

### Data analysis

Alleles were assigned based on the sizes of PCR amplifications that were generated by each pair of SSR primers using GeneMarker software version 1.31 with a binning procedure. PCR amplification with an identical size generated by the same pair of primers was considered as an allele. Multilocus genotype for each isolate was formed by joining the alleles at each SSR locus in the same order across all primers and clones were determined by GenClone 2.0 (Arnaud-Haond and Belkhir, 2007). All subsequent analyses except the Shannon index were calculated with clone-corrected data by using only one representative isolate of the same genotype (McDonald, 1997).

Isolates were hierarchically grouped into “field” and “regional” subpopulations. Isolates collected from the same field at the same point in time were classified as a field subpopulation and those from different fields within the same cropping region were considered as a regional subpopulation. Based on climatic conditions and farm practices, potato production in China is divided into four cropping regions (Jansky et al., 2009)—the Northern Single-cropping Region (NSR), the Central Double-cropping Region (CDR), the Southwestern Multiple-cropping Region (SMR), and the Southern Winter-cropping Region (SWR).

Genetic variation in the subpopulations was evaluated by gene diversity (Nei, 1973), allele number, and standardized Shannon index (Grünwald et al., 2003). Allele number and gene diversity were estimated by Popgene v. 1.32 (Yeh et al., 2000) and the Shannon index was calculated using an Excel spreadsheet. The difference in genetic variation between the regional populations was evaluated by bootstrap (Gao et al., 2017) with 100 replicates using the Resampling Stats add-in package for Excel (Blank et al., 2001). For each bootstrap replication, gene diversity was recorded from a random sample of 40 multilocus genotypes (the actual number of multilocus genotypes in SWR population). The mean and variance of gene diversity were calculated and used for a *t*-test. The model-based Bayesian method implemented in Structure v. 2.3.4 (Pritchard et al., 2000) was used to evaluate the admixture of the fungal isolates by grouping them into K (*K* = 1, 2 ……) distinct clusters regardless of geographical origins while minimizing the gametic phase disequilibrium between loci within clusters (Pritchard et al., 2000; Hubisz et al., 2009). The Monte Carlo Markov chain (MCMC) was run for 1,200,000 iterations with a 200,000 burn-in period, with *K* ranging from 1 to 10 and 10 independent replications for each *K*. Δ*K* computed from Structure Harvester (http://taylor0.biology.ucla.edu/struct_harvest/) was used to determine the optimal value of *K* using an admixture model (Evanno et al., 2005).

Pair-wise unbiased genetic distance (Nei, 1978) between the 17 field subpopulations was estimated using the Microsatellite Analyser (MSA) program (Dieringer and Schlötterer, 2003). The results were visualized by principal coordinates analysis (PCoA) with GenAlEx6 (Peakall and Smouse, 2006) and a neighbor-joining (Ali et al., 2014) tree using Phylip (Felsenstein, 2002). The robustness of the NJ tree was tested by 1,000 bootstraps of original data with a microsatellite analyser (MSA) (Dieringer and Schlötterer, 2003). The genetic distance was also used to evaluate the association between fungal isolates and their geographical origin (region) and the trees were displayed using Mega 5 (Tamura et al., 2011).

Spatial distribution of genetic variation in the *A. alternata* subpopulations was estimated by an analysis of molecular variance (AMOVA) with GenAlEx6 and genetic differentiation (Nei, 1973) was estimated with Popgene 1.32 (Yeh et al., 2000) using weighted gene frequencies for small and unequal sample sizes (Nei, 1973; Nei and Chesser, 1983). Isolation-by-distance (Slatkin, 1993) was evaluated by estimating the association between the extent of gene flow and the natural logarithm of physical distance in the fungal populations (Zhan et al., 2003). The physical distance between pair of subpopulations was estimated using the geographic coordinates of locations from which the subpopulations were sampled (Zhu et al., 2015).

The amount and direction of gene flow among regional subpopulations were measured by Migrate v. 3.6.1 (Beerli and Felsenstein, 2001). The computations were carried out using coalescent theory under the model-based Bayesian method. The extent of gene flow within and between the regional subpopulations was also evaluated by F-statistics (Harrison et al., 1965) assuming a migration drift equilibrium (Palsbøll et al., 2007).

All field subpopulations were evaluated to determine whether there was an excess (a recent population bottleneck) or deficit (a recent population expansion) in genetic diversity relative to the observed value with Bottleneck 1.2 (Piry et al., 1999). The Sign and Wilcoxon tests were used to determine the excess or deficit in gene diversity by comparing the observed gene diversity (*H*) with expected gene diversity (*H*_EQ_) based on the infinite alleles model, two-phase mutation models (TPM) and SMM (70% SMM and 30% IAM) (Cornuet and Luikart, 1996; Piry et al., 1999; Tsui et al., 2012).

## Results

### Genetic variation in *A. alternata*

Gene diversity ranged from 0.25 to 0.64 with an average of 0.38 and allele number ranged from 2.13 to 5.13 with an average of 3.58 in the 17 field subpopulations (Table 1). Gene diversity in the two field subpopulations (FZH and CHL) from the winter cropping region were 0.64 and 0.56. At the regional level, gene diversity ranged from 0.35 in NSR to 0.63 in SWR with an average of 0.45. Gene diversity in SWR was significantly higher than that in other regions (*p* < 0.001). The overall gene diversity and number of alleles detected were 0.42 and 7.25, respectively, when all isolates from the study were pooled.

A total of 253 distinct multilocus genotypes were detected in the 725 *A. alternata* isolates assayed with the eight pairs of SSR primers (Table 1). Among these multilocus genotypes, 134 were detected only once and 103 were detected in more than one field. The most common multilocus genotype was detected 24 times and the most widespread multilocus genotype was detected in nine fields across three regions. The number of multilocus genotypes ranged from 5 in KMG2 to 52 in CIF with an average of 29.18 in the field subpopulations and from 40 in SWR to 232 in NSR with an average of 124 in the regional populations. No multilocus genotypes were shared between KMG1 and KMG2, the two populations sampled from the same field in one-year interval. Normalized Shannon index values ranged from 0.84 to 1.00 with an average of 0.90 in the field subpopulations and from 0.73 to 0.88 with an average of 0.83 in the regional populations. At the field level, subpopulations from CGD, DAT, and QJG had the highest Shannon index values while subpopulations from CHL had the lowest. At the regional level, populations for the CDR and NSR had the highest and lowest Shannon index values, respectively. The overall Shannon index was 0.78 when all isolates were combined into a single population (Table 1).

### Spatial population genetic structure in *A. alternata*

**Figure 2:**
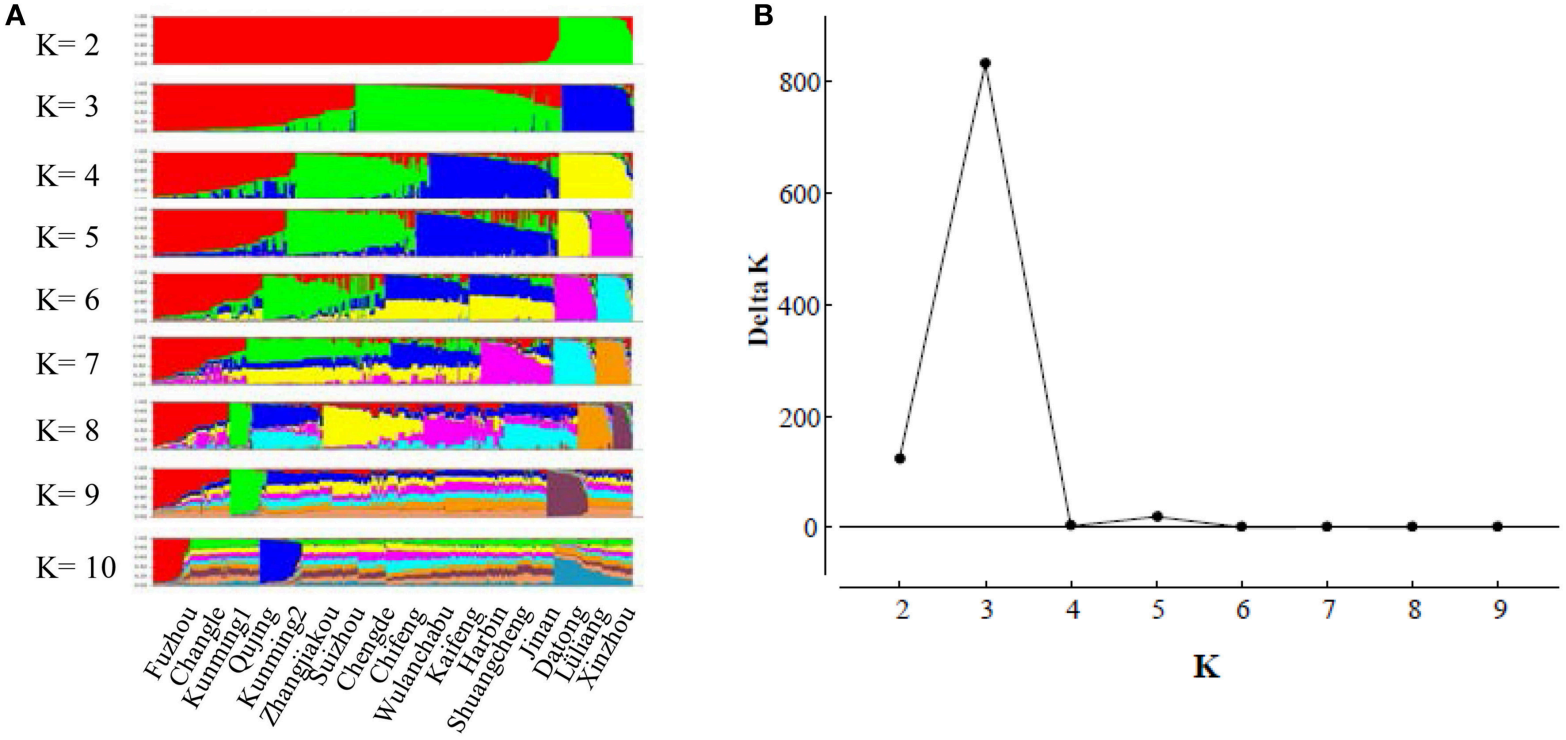
The evaluation of population admixture in the *Alternaria alternata* population from China using Structure. **(A)** The distribution of isolate assignment for number of clusters (*K*) ranging *K* from 2 to 10; **(B)** The estimated Delta *K* (Δ*K*) for number of clusters ranging from 2 to 9.

**Figure 3:**
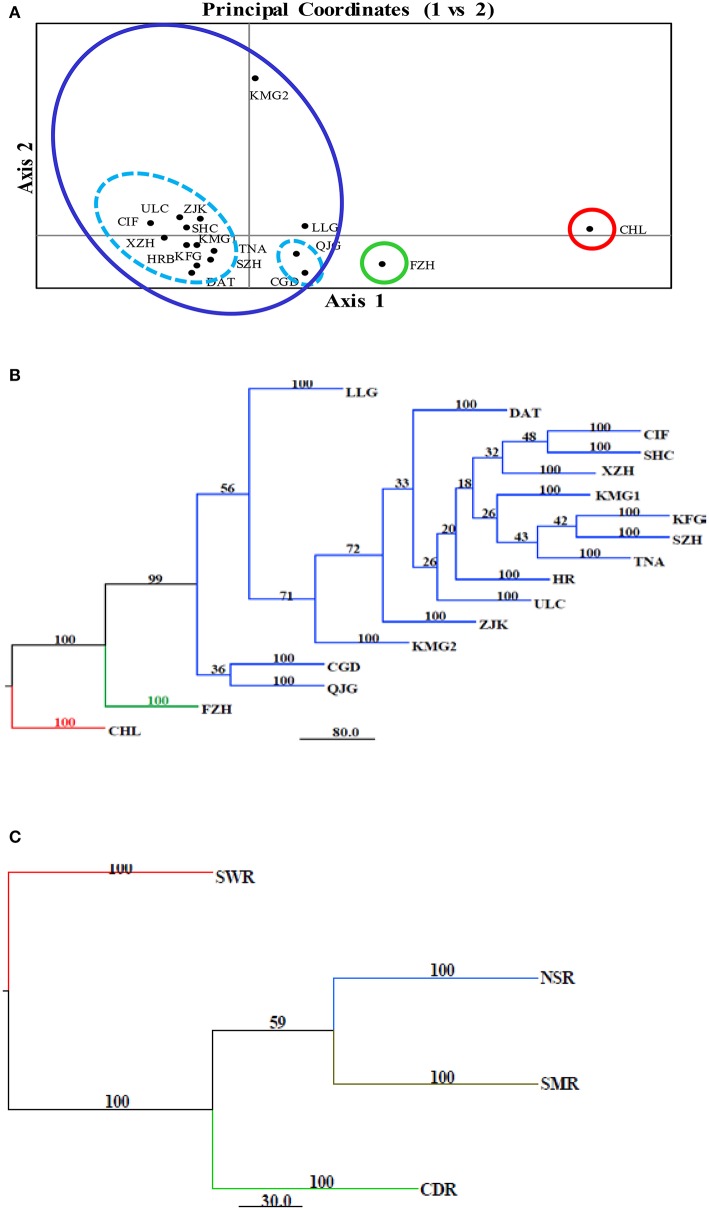
Cluster analyses of 17 *Alternaria alternata* subpopulations from China. **(A)** Principal Coordinates Analysis of the pathogen populations using Nei's genetic distance estimated with GenAlEx. The first and second coordinates accounted for 77.25 and 13.27% of the total variance, respectively; **(B)** Neighbor joining tree of the 17 *Alternaria alternata* subpopulations reconstructed with the program implemented in the Phylip package; **(C)** Neighbor joining tree of four regional subpopulations reconstructed with the program implemented in the Phylip package. Bootstrap support was generated by 1,000 resampling of original data using Microsatellite Analyzer.

**Figure 4:**
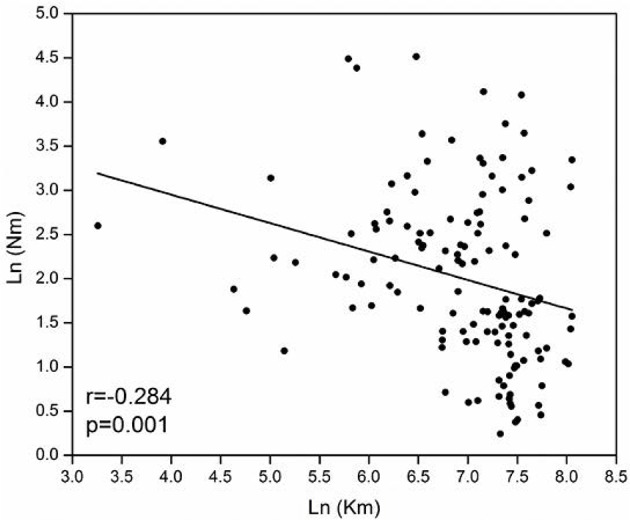
Association between geographical distance and gene flow for 17 *Alternaria alternata* subpopulations collected from China.

**Figure 5:**
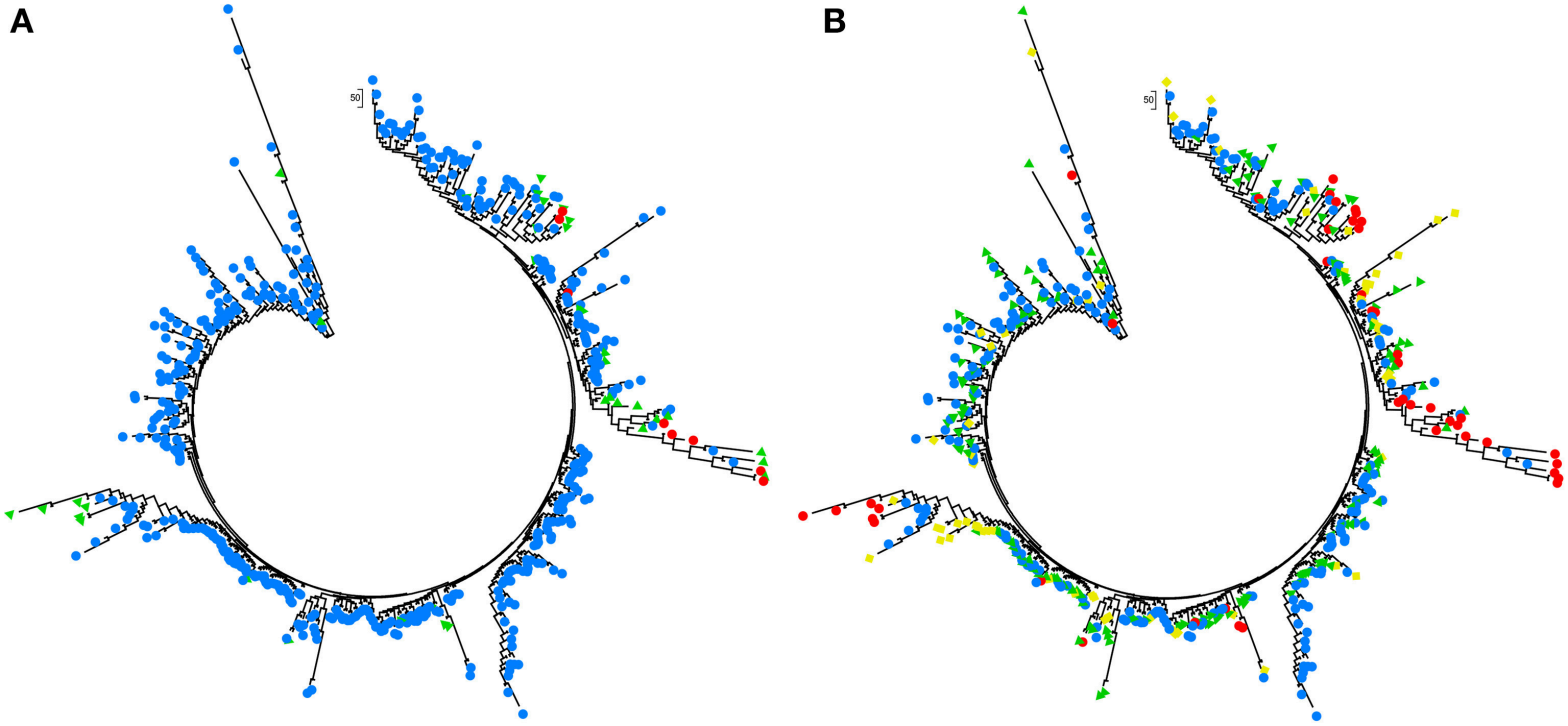
Analyses of phylogenetic association in the 253 *Alternaria alternata* multilocus genotypes detected from the 725 isolates sampled from China with Nei's genetic distance calculated with GENALEX 6.5. **(A)** The association between multilocus genotypes and the clusters assigned by admixture analysis with Structure. Colors represented the multilocus genotypes isolates from different clusters and **(B)** the association between multilocus genotypes and their geographic origins. The phylogenetic trees were reconstructed using Mega 5 and colors represent different clusters or geographic origins. Red, yellow, green, and blue represented the multilocus genotypes isolates from SWR, SMR, CDR, and NSR, respectively.

The model-based Bayesian clustering algorithms implemented in Structure generated different population structure patterns with increasing *K*-value from 2 to 10 (Figure 2A). The optimized population structure patterns were retained when the isolates, taking no account of the sampling spatial location, were assigned into three clusters (Figure 2B). Similarly, PCoA also grouped the 17 field subpopulations into three clusters. CHL and FZH subpopulations were grouped into two different clusters, while the other 15 subpopulations were grouped into a single cluster (Figure 1A). In this PCoA analysis, the first and second axes accounted for 77.25 and 13.27% of the total variance, respectively. A NJ tree reconstructed from the 17 field subpopulations produced the same clusters as the PCoA. CHL and FZH from SWR formed two distinct clusters with 100% bootstrap support (Figure 3B). The other 15 field subpopulations from NSR, CDR, and SMR formed a single major cluster with 99% bootstrap support (Figure 3B). At the regional level, the SWR population grouping formed an independent cluster separated from the other three cropping regions (NSR, CDR, and SMR) with 100% bootstrap support (Figure 3C). The differentiation among years, among cropping regions, among subpopulations within regions and among individuals within subpopulations contributed to 6.27, 3.14, 2.48, and 88.11% of total variation, respectively; all of these contributions were significantly higher than zero (*P* < 0.001, Table 2). There was a negative correlation between gene flow and geographic distance [*r*_(134)_ = −0.284, *p* = 0.001] among field subpopulations (Figure 4), but no genetic association among fungal isolates and their geographical origin (Figure 5).

**Table 2:** Analysis of molecular variance (AMOVA) for the 17 field subpopulations of *Alternaria alternata* sampled from four cropping regions in China.

	**DF**	**SS**	**Est. Var**.	**%**	***p*-value**
Among years	1	983.71	7.80	6.27	<0.001
Among regions	3	1826.94	3.90	3.14	<0.001
Among fields	13	2565.64	3.09	2.48	<0.001
Within fields	479	51369.20	109.58	88.11	<0.001
Total	496	56745.49	124.37	100.00	

### Amount and direction of gene flow among regional *A. alternata* populations

Classical estimates of gene flow assuming an island model ranged from 4.37 to 11.03 (Table 3) within cropping regions with an average of 8.33 and from 3.56 to 37.58 among cropping regions with an average of 18.15 (Table 4). Substantial gene flow was also detected among the four cropping regions using a coalescence approach, with the estimated number of migrants per generation ranging from 44.33 to 855.70 (Figure 1B). NSR was the largest source population donating 21.74 (269.41–247.67), 676.03 (855.70–179.67), and 661.52 (714.59–53.07) net migrants each generation to CDR, SMR, and SWR; while SWR was the largest sink population receiving 263.63 (374.34–110.71), 246.01 (290.34–44.33), and 661.52 (714.59–53.07) net migrants each generation from CDR, SMR, and NSR, respectively (Figures 1B,C). CDR was the second largest source population donating 85.98 and 263.63 net immigrants per generation to SMR and SWR; while SMR was the second largest sink population receiving 676.03 and 85.98 net immigrants each generation from NSR and CDR, respectively. The number of migrants exchanged per generation between the NSR and CDR populations was almost the same.

**Table 3:** Classical estimates of population differentiation and average migrants per generation (Nm) in the four regional subpopulations of *A. alternaria alternata* sampled from China.

**Cropping regions**	***G*_ST_**	***N*m**
CDR	0.04	11.93
NSR	0.08	5.97
SMR	0.10	4.37
SWR	0.04	11.03

**Table 4:** Pair-wise population differentiation (Harrison et al.) and average number of migrants per generation (Nm) estimated from the classical island model between the four regional subpopulations of *A. alternata* sampled from China.

**Cropping regions**	**CDR**	**NSR**	**SMR**	**SWR**
CDR	—	37.58	31.72	4.59
NSR	0.01	—	26.48	3.56
SMR	0.02	0.02	—	4.95
SWR	0.10	0.12	0.10	—

*Values above the diagonal are Nm and below the diagonal are G_ST_*.

At the field subpopulation level, the allele frequency in 9/17 (52.94%) subpopulations (SZH, ULC, KFG, HRB, SHC, JNN, DAT, LLG, and XZH) showed a normal L-shaped distribution with no significant excess or deficit in gene diversity (Table 5) under all three models while two (11.76%) subpopulations (CHL and CGD) showed a significant excess under TPM (Table 5). Six subpopulations (FZH, KMG1, QJG, KMG2, ZJK, and CIF) were found to have a significant deficit in gene diversity in one of the three models but significance was not attained by the Wilcoxon's test (Table 5). At the regional level, two populations were found to have a significant deficit under the mixed model but again were not significant according to Wilcoxon's test (Table 5).

**Table 5:** Comparison of observed gene diversity (H) with expected gene diversity (H_EQ_) at mutation-drift equilibrium calculated from the observed number of alleles under IAM, SMM, and TPM for the 17 *Alternaria alternata* subpopulations.

**Subpopulation**	**Mutation model**	**D/E^a^**		
		**IAM^b^**	**TPM^c^**	**SMM^d^**
FZH	SMM (*p* = 0.990^ns^)	1/7^ns^	4/4^ns^	7/1^**^
CHL	TPM (*p* = 0.004^***^)	0/7^*^	0/7^*^	3/4^ns^
KMG1	IAM (*p* = 0.578^ns^)	3/3^ns^	4/2^ns^	5/1^*^
QJG	IAM (*p* = 0.973^ns^)	6/1^*^	6/1^*^	6/1^*^
KMG2	SMM (*p* = 0.998^ns^)	3/5^ns^	5/3^ns^	7/1^**^
ZJK	SMM (*p* = 0.994^ns^)	4/4^ns^	5/3^ns^	7/1^**^
SZH	IAM (*p* = 0.727^ns^)	4/4^ns^	4/4^ns^	5/3^ns^
CGD	IAM (*p* = 0.063^ns^)	1/7^*^	1/7^ns^	5/3^ns^
CIF	TPM (*p* = 0.875^ns^)	4/4^ns^	4/4^ns^	6/2^**^
ULC	IAM (*p* = 0.500^ns^)	2/3^ns^	2/3^ns^	2/3^ns^
KFG	IAM (*p* = 0.727^ns^)	4/4^ns^	4/4^ns^	4/4^ns^
HRB	IAM (*p* = 0.629^ns^)	4/4^ns^	4/4^ns^	5/3^ns^
SHC	IAM (*p* = 0.809^ns^)	5/3^ns^	5/3^ns^	5/3^ns^
JNN	IAM (*p* = 0.578^ns^)	4/4^ns^	4/4^ns^	5/3^ns^
DAT	IAM (*p* = 0.578^ns^)	3/5^ns^	6/2^ns^	6/2^ns^
LLG	IAM (*p* = 0.273^ns^)	2/6^ns^	5/3^ns^	5/3^ns^
XZH	IAM (*p* = 0.078^ns^)	1/4^ns^	1/4^ns^	1/4^ns^
CDR	SMM (*p* = 0.986^ns^)	4/4^ns^	4/4^ns^	6/2 ^ns^
NSR	SMM (*p* = 0.998^ns^)	4/4^ns^	4/4^ns^	7/1^**^
SMR	SMM (*p* = 0.990^ns^)	5/3^ns^	6/2^ns^	8/0^**^
SWR	SMM (*p* = 0.125^ns^)	1/7^ns^	1/7^ns^	2/6^ns^

a The number of loci showing a deficit/excess of gene diversity. Significance estimates of excess or deficiency across loci were obtained using the one-tailed Wilcoxon test and the Sign test.

b Infinite allele model.

c Stepwise mutation model.

d Two-phase mutation models.

* p ≤ 0.05,

** p ≤ 0.01,

ns *p > 0.05*.

## Discussion

Due to habitat fragmentation, seasonality and regular changes of cultural practices, local extinctions, and recolonizations are very common in plant pathogens in agricultural systems (Stukenbrock and McDonald, 2008). The extinctions and recolonizations are expected to occur more often in the early blight disease of potato in China. In the Southern China such as Fuzhou, potato is rotated with rice every year while in the Northern China such as Harbin and Wulachabu, temperature in winter can reach as low as <–30°C (Zhu et al., 2015), largely preventing the carryover of *A. alternata* from seasons to seasons in the same fields. Under these scenarios, pathogen populations in the fields are regularly replaced by founders from infected plant materials or nearby regions, supported by the high differentiation in the two pathogen populations sampled from the same field (KMG1 and KMG2) at different years and quick population turn-over of the pathogen (Vandecasteele et al., 2017). Frequent founder effect resulting from severe reductions in population size after each cycle of recolonization can not only reduce the genetic diversity of local subpopulations directly but also increases the chance that genetically related individuals mate with each other (inbreeding), further reducing the genetic variation and ecological resilience of these pathogen subpopulations. It is also expected high genetic differentiation exists among subpopulations of plant pathogens in agricultural systems attributable to high levels of genetic drift generated by stochastic changes in allele frequencies during recolonization events (Giraud et al., 2010; Rieux et al., 2013). Against to these expectations, many pathogens of agricultural crops show high genetic variation within subpopulations and low spatial differentiation among subpopulations for neutral markers (e.g., Ali et al., 2014). These patterns of population genetic structure are also found in the current study (Tables 1–4, Figures 2, 4), These results consist that populations of *A. alternata* and many other plant pathogens in agricultural systems (Zhan et al., 2003; Castorani et al., 2017) are genetically linked by regular gene flow, preventing substantial loss of genetic variation within subpopulations and accumulation of genetic differentiation among subpopulations. In this respect the pathogen displays a unique characteristic of metapopulation genetic structure (Hanski, 1998).

The metapopulation genetic structure of *A. alternata* infecting potato in China is also supported by admixture, principal coordinate, phylogenetic and demographic analyses of the pathogen. All subpopulations except FZH and CHL formed into a single major cluster by admixture and principal coordinate analyses (Figures 2, 3) and isolates from each subpopulation were randomly distributed in clusters and cropping regions (Figures 2, 3, 5). With the exception of the CHL subpopulation, no bottlenecks were detected in any subpopulations at either the field or regional level (Table 5). FZH and CHL are two subpopulations collected from SWR where seed tubers are primarily imported from other parts of China. It is likely the two subpopulations were emerged from very recent admixture events, as commonly detected in hybrid zones when subpopulations come into contact via dispersal or from sharing a common border (Vähä and Primmer, 2006). Recent admixture coupled with the small sample size (only 11 isolates) may also explain the detection of population contraction in CHL.

These results fit with the concept that nationwide dispersal of the pathogen reproductive unit creates opportunities for subpopulation assemblages and demographic stability and may contribute to quick adaptation of many plant pathogens to management approaches such as the deployment of major resistance genes and application of site-specific fungicides. Isolation-by-distance analysis reveals negative correlation between the extent of gene flow and geographic distance (Figure 4). In nature, both natural selection for local adaptation resulted from habitat heterogeneity and restricted gene flow caused by geographic barriers can generate isolation-by-distance patterns of genetic variation (Feng et al., 2016; Petkova et al., 2016). But in the current study, we believe that the observed isolation-by-distance is due to geographic barriers because the molecular markers we applied are selectively neutral (Meng et al., 2015b). Subpopulations that are geographically closer are expected to exchange more genetic information over time and should have a tendency to be more alike at neutral markers (Hall, 2006; Baumgartner et al., 2010; Margos et al., 2012). The finding of isolation-by-distance indicates natural gene flow is high (Cornille et al., 2015) in the evolutionary history of the pathogen.

Though negative association between gene flow and geographic distance, the model only accounts for <10% of variation (*R*^2^ = 0.284^2^ = 0.08, Figure 4), suggesting a weak pattern of isolation-by-distance in the pathogen. This may partly reflect human-mediated dispersals mitigating the impact of geographic barriers on the generation of spatial aggregation in the pathogen populations. Human-mediated dispersal can occur through anthropogenic activities such as commercial trading of plant materials. In China, large quantities of potato products are moved around the country annually either as seed tubers or foods and biological contaminations of these products are not well regulated. The loose phytosanitation procedure facilitates the massive movement of the pathogen gene pool across long distance regularly (Zhu et al., 2015). We have several lines of evidence to support the hypothesis of human-mediated dispersal connecting subpopulations of *A. alternata*.

Theoretically, when species expand their ranges to new habitats, correlations between genetic and geographic distances are low at first and then increase gradually until reaching a plateau (Slatkin, 1993; Matsuda et al., 2015; Short et al., 2015). This model predicts that the pattern of isolation-by-distance should be first attained, and be more obviously apparent, at short geographic distances. However, no such patterns were observed when we regrouped the subpopulations into four groups according to their geographic distances (<3,500, 2,500, 1,500, and 500 km) and re-calculated the correlation between gene flow and physical distance (data not shown), indicating anthropogenic disturbances may have occurred in the pathogen.

Higher genetic differentiation as a result of lower gene flow was observed within cropping regions than among cropping regions in the current study (Tables 3, 4). Similar patterns of spatial population genetic structure have also been found in other agricultural plant pathogens (Rieux et al., 2013; Taole et al., 2015), suggesting they are not unique to *A. alternata*. These results are counter intuitive and cannot simply be explained by natural dispersal, which is expected to create higher genetic similarity (lower genetic differentiation) among geographically closer subpopulations. In China, potatoes in the same cropping regions are usually planted and harvested at similar times and exchange of potato products as planting material or food is much less common within than among cropping regions. As a result, gene flow within cropping regions is much lower than among cropping regions due to the reduced human-mediated dispersal of pathogen through infected tubers in the former.

Human-mediated dispersal of *A. alternata* is also supported by the directions of gene flow estimated by a coalescent approach (Figure 1) and the pattern of gene diversity detected in cropping regions (Table 1). The estimated directions of gene flow are concordant with movements of potato products but are counter to prevailing wind patterns during the main growing seasons. In the country, potatoes are mainly produced in NSR and SMR and shipped from north to south and west to east either as seeds or foods while winds move from east to west and south to north during the main potato growing season. Of all regions NSR imports the fewest potatoes while SWR imports the most from NSR, leading to the lowest gene diversity in the former but the highest gene diversity in the latter.

Our results clearly indicate that human-mediated dispersal plays an important role in the generation and dynamics of metapopulation genetic structure in *A. alternata* as reported in other pathogen species (Linde et al., 2009; Talbi et al., 2010). Metapopulation structure extends the lifespan of plant pathogens from a single, transient subpopulation evolved for a particular ecological niche to the network of all subpopulations adapted across multiple ecological niches (Wahlberg et al., 2002; Postma and van Noordwijk, 2005). In agricultural ecosystems, metapopulation structure of plant pathogens increases the adaptive capacity of local subpopulations (Hanski and Ovaskainen, 2000) as a result of boosted genetic variation attributable to the continuous influx of new genetic information from neighboring subpopulations (Levins, 1969; Laine, 2005; Numminen et al., 2016) or reduced inbreeding depression (Richards, 2000), facilitating the replacement of subpopulations with superior genes or gene combinations (Flaxman et al., 2014) such as novel fungicide resistance and virulence factors and contributing to the quick breakdown of many disease management approaches. In this case, implementing a strict quarantine regulation both within and beyond political borders may be required to reduce the evolutionary potential of pathogens for a sustainable plant disease management.

## Data archiving statement

Data for this study will be available at the Dryad Digital Repository after manuscript is accepted for publication.

## Author contributions

J-WM and D-CH: Collected pathogen isolates, generated and analyzed the data, and wrote the manuscript; WZ, L-NY, E-JW, J-HX, and L-PS: Collected pathogen isolates and generated the data; JZ: Conceived and designed the experiments, analyzed the data, and wrote the manuscript. All authors reviewed the manuscript.

### Conflict of interest statement

The authors declare that the research was conducted in the absence of any commercial or financial relationships that could be construed as a potential conflict of interest.
